# Management of Non-Colorectal Digestive Cancers with Microsatellite Instability

**DOI:** 10.3390/cancers13040651

**Published:** 2021-02-06

**Authors:** Mojun Zhu, Zhaohui Jin, Joleen M. Hubbard

**Affiliations:** Department of Medical Oncology, Mayo Clinic, Rochester, MN 55905, USA; Jin.Zhaohui@mayo.edu (Z.J.); Hubbard.Joleen@mayo.edu (J.M.H.)

**Keywords:** microsatellite instability, checkpoint, gastrointestinal cancer

## Abstract

**Simple Summary:**

Microsatellite instability (MSI) is an established predictive biomarker for immune checkpoint inhibitors with potential prognostic value in different types of tumors. Its prevalence and clinical utility vary in gastrointestinal cancers. In this review, we will discuss the role of MSI status in the management of non-colorectal cancers of the digestive system and address ongoing research work and mechanistic rationale(s) for future studies in this field.

**Abstract:**

Microsatellite instability (MSI) is a hallmark of genetic predisposition to DNA damage. It arises from either germline or somatic events leading to impaired function of the mismatch repair system. It can be detected via genetic sequencing or immunohistochemistry with relatively high concordance rates. The presence of MSI in a tumor reflects a high neoantigen load and predicts favorable treatment response to immune checkpoint inhibitors (ICIs). In gastrointestinal cancers, MSI is a predictive biomarker for ICIs with potential prognostic impact but its clinical utility varies widely depending on tumor type. This may be explained by the complexity of tumor microenvironment as highlighted by recent translational studies. In this review, we will discuss the predictive and prognostic value of MSI status in non-colorectal cancers of the digestive system, important clinical trials involving ICIs and potential strategies to overcome resistance to immunotherapy.

## 1. Introduction

Microsatellites are short tandem repeats of 1–6 base pairs often found in the non-coding regions of DNA; replication errors in these areas are mainly corrected by the mismatch repair (MMR) system [1]. Inactivation or suppressed expression of MMR proteins gives rise to microsatellite instability (MSI), a hallmark of genetic predisposition to DNA damage. This could occur as a result of genetic mutation or promoter methylation due to epigenetic events secondary to either germline or sporadic changes [2,3,4]. For instance, Lynch syndrome is an autosomal dominant germline disorder marked by impaired MMR, predisposing its carriers to multiple types of malignancies [4]. 

MSI is divided into high (MSI-H), stable (MSS) and low (MSI-L) based on the frequency of changes in alleles, which can be quantified by polymerase chain reaction (PCR) amplification or next-generation sequencing (NGS). MSI-H tumors have histopathological and clinical characteristics distinct from MSS and MSI-L tumors, whereas MSS and MSI-L tumors behave similarly [5,6]. For PCR-based testing, DNA is extracted from paired tumor and normal tissue and amplified at five chosen markers (BAT25, BAT26, D5S346, D2S123 and D17S250); alterations in the repeat length of each marker are compared between tumor and normal tissue [6]. MSI-H tumors are defined as having instability in ≥2 markers, MSI-L tumors have instability in one marker and tumors without instability are MSS [6]. If more than fiver markers are tested, MSI-H tumors will be defined as having instability in ≥30% of the loci studied [6]. NGS-based testing is becoming more popular, but the performance of this test highly depends on the selection of microsatellite loci. Compared to PCR-based detection, NGS does not require matched normal tissue and may be more sensitive [7]. 

Deficient MMR (dMMR) entails a process whereby dysfunctional MMR proteins at the germline or somatic level lead to the MSI-H phenotype. This can be detected via immunohistochemistry (IHC) that assesses the expression of four MMR proteins (i.e., MLH1, PMS2, MSH2 and MSH6). MSI-H and dMMR have, thus, been used interchangeably to describe the same phenotype with high concordance between diagnostic tests (i.e., PCR, NGS and IHC) [5,8,9,10]. However, it is important to acknowledge the discrepancy. About 5–11% of MSI-H tumors are tested normal by IHC, as they have nonfunctional MMR proteins which are not evaluated by the current IHC method [11]. Vice versa, loss of MSH6 protein that is detectable by IHC can be associated with MSS or MSI-L tumors [12]. 

MSI-H tumors were first noted to be enriched by tumor-infiltrating lymphocytes (TILs) in colorectal cancer (CRC) [13], making them attractive targets for immune checkpoint inhibitors (ICIs) that predominantly induce tumor cell killing by reinvigorating the adaptive immune system. T-cell signaling plays an indispensable role in orchestrating the adaptive immune response. Apart from the antigen-specific signaling through T-cell receptors, stimulation through CD28, a co-stimulatory cell surface receptor on T cells, via antigen-nonspecific mechanisms further augment T-cell activity [14]. Co-inhibitory receptors (e.g., programmed death-1 [PD-1], cytotoxic T lymphocyte associated protein-4 [CTLA-4]) were discovered along the unearthing of CD28 [15,16,17]. ICIs are antibodies that are designed to block co-inhibitory receptors or their ligands and downregulate immunosuppression. They have been approved for different indications in gastrointestinal (GI) cancers (Table 1). 

Efficacy of ICIs in MSI-H tumors was first noted in a phase 1 study of nivolumab (a PD-1 inhibitor), which enrolled 39 patients with heavily treated solid tumors [18,19,20]. Only one patient with MSI-H CRC had a complete response to nivolumab that was maintained for three years without recurrent disease, providing strong evidence to support the hypothesis that MSI-H tumors carry high neoantigen load and stimulate the immune system more effectively than MSS/MSI-L tumors [21,22,23]. Le et al. subsequently showed that pembrolizumab (another PD-1 inhibitor) led to radiographic responses in ≥30% of patients with MSI-H tumors [24], leading to the first tissue/site agnostic approval for its use in MSI-H/dMMR solid tumors refractory to prior systemic therapies by the U.S. Food and Drug Administration (FDA). Nivolumab with or without ipilimumab (a CTLA-4 inhibitor) were also approved for the treatment of MSI-H CRC that has progressed on chemotherapy based on the CheckMate142 study which reported response rates (RRs) of 31% with nivolumab monotherapy and 55% with the combination [25,26].

The prevalence of MSI-H varies in GI malignancies. It occurs with relatively high frequency in CRC (10–15%) [3] and gastric cancer (~10%) [27]. It was reported to be less than 5% in hepatocellular carcinoma, cholangiocarcinoma, esophageal and pancreatic adenocarcinoma, respectively [28,29,30]. Universal screening for MSI is recommended at the diagnosis of CRC by most guidelines but not yet in non-CRC GI cancers. With the dramatic response of MSI-H tumors to immunotherapy, MSI testing has, however, increased significantly in all solid tumors. The FDA has recently approved pembrolizumab as a first-line therapy for MSI-H/dMMR metastatic CRC based on the phase 3, randomized KEYNOTE-177 study, which reported an improved RR (43.8% versus 33.1%) and progression-free survival (PFS, median 16.5 vs. 8.2 months, hazard ratio [HR] 0.60, 95% confidence interval [CI] 0.45–0.80, *p* = 0.0002) with pembrolizumab monotherapy compared to the physicians’ choice of 5-fluorouracil-based chemotherapy [31,32]. Given the success of ICIs in frontline therapy for metastatic CRC, there is a surge of clinical trials aiming to improve its use for the treatment of non-CRC GI cancers. We will review major clinical trials involving ICIs in non-CRC GI cancers and the current state of research focusing on this area (Table 2). 

## 2. Landmark Studies and Key Concepts in MSI-H Non-CRC Cancers of the Digestive System

In 2017, Le et al. published a retrospective study based on data from five clinical trials (KEYNOTE-012, 016, 028, 158 and 164) and established the efficacy of pembrolizumab in MSI-H tumors [23]. For the final market application of pembrolizumab, 149 patients with MSI-H tumors of 15 different tumor types (90 CRC and 59 non-CRC) were identified with an RR of 39.6% (complete response rate of 7%) and more than two-thirds of patients maintained responses for ≥6 months [63]. 

Besides MSI, tumor mutational burden (TMB) and PD-L1 are important biomarkers in the era of ICIs. Similar to MSI, TMB reflects neoantigen load and high TMB (TMB-H) predicts responses to ICIs [21,22,23]. TMB is usually quantified by exome sequencing or NGS of tumor tissues but the cutoff for TMB-H varies among different studies and testing platforms [34,64]. Based on the phase 2 KEYNOTE-158 study, 29% of 102 patients with TMB-H (≥10 mut/Mb) tumors that had progressed on prior therapy responded to pembrolizumab monotherapy (median duration of response [DOR] not reached [NR]) and the FDA granted accelerated approval to its use for the treatment of solid tumors with TMB ≥10 mut/Mb (determined by an FDA approved test) that is refractory to prior treatment [34].

PD-L1 staining of tumor cells and/or TILs, on the other hand, is perhaps more reflective of immune responses and it predicts favorable outcomes to ICIs in certain tumor types only. It is evaluated by IHC assays; PD-L1 combined positive score (CPS) using the PD-L1 IHC 22C3 pharmDx (Agilent, Carpinteria, CA, USA) is commonly applied to GI cancers [65]. Notably, PD-L1 CPS accounts for both PD-L1 positive tumor cells and immune cells and seems highly reproducible in gastric cancer with higher CPS indicating increased likelihood of responding to pembrolizumab [66]. In the phase 2 KEYNOTE-59 study, 15.5% of 148 patients with PD-L1 CPS ≥ 1 gastric cancer that had progressed after 2 or more prior lines of therapy had radiographic responses to pembrolizumab with a median DOR of 16.3 months (range 1.6–17.3 months) [37], and it was approved by the FDA for this indication. 

The presence of MSI-H/dMMR, TMB-H and PD-L1 overlap in solid tumors with different frequencies [10,34,67] and their predictive power has not been compared in a systematic fashion (Table 3). Reproducibility and reliability of these test results remain a significant problem too. Intertumoral heterogeneity (e.g., primary vs. metastatic tumors), specimen preservation conditions (e.g., cryopreservation vs. paraffin embedding), technique variations across different platforms (e.g., FoundationOne CDx vs. Tempus Xt) and inconsistent scoring systems (e.g., CPS vs. others) confound the interpretation of test results. Overall, there is a lack of data to guide testing choice. Research is ongoing to harmonize the results and discover better biomarkers and technologies, which will help to address these challenges. 

## 3. Gastric and Gastroesophageal Adenocarcinoma

Gastric cancer (GC) consists of malignancies arising from the stomach and the gastroesophageal junction (GEJ). Traditionally, GEJ adenocarcinoma is classified based on the anatomic Siewert system, and Siewert type III GEJ adenocarcinoma, located below 1 cm above the gastric cardia, are grouped within GC [81]. GC is commonly divided into four subtypes based on a molecular classification system: microsatellite unstable, Epstein-Barr virus (EBV) positive, genomically stable and chromosomal instable tumors [82]. MSI-H and EBV-positive GC are associated with lymphocyte-rich GC, which has a better prognosis than other types of GC [83]. It was proposed that the presence of lymphocytes in tumor tissues reflects effective host immune response against malignancy and confers survival benefits.

The prevalence of MSI-H was reported ranging from 4–25% in gastric and GEJ adenocarcinoma without significant differences between Asian and Caucasian population [27,37,38,39,67,82,84]. MSI-H GC is diagnosed at older age, more common in female and non-cardia GC and with less lymph node involvement [82,84,85]. Data suggest that MSI-H GC is associated with improved survival, but these studies were retrospective in nature and confounded by inter-study heterogeneities [84,86,87,88,89]. Based on a meta-analysis of four major randomized clinical trials, MSI-H GC was independently associated with improved disease-free survival (five-year DFS rates, 71.8% vs. 52.3%, *p* < 0.001) and overall survival (five-year OS rates, 77.5% vs. 59.3%, *p* < 0.001) [88]. Although one study reported similar survival, patients with surgically resected MSI-H GC did not derive survival benefits from perioperative chemotherapy, indicating that MSI may be a predictive biomarker for resistance to chemotherapy [88,89]. 

MSI-H GC was also found to have increased immune cell infiltration and PD-L1 expression [90], hinting that improved survival of these patients may be secondary to upregulated immunosurveillance. In turn, MSI-H tumors may be more sensitive to ICIs. In the relapsed or refractory setting, 45.8% of 24 patients with MSI-H GC had radiographic responses to pembrolizumab in the single-arm, phase 2 KEYNOTE-158 study [35]; similar responses (RR 50%) were also observed with MSI-H tumors in the gastric cancer cohort of the phase 1b KEYNOTE-12 study [36]. Another South Korea-based, phase 2 study reported an RR of 85.7% in seven MSI-H metastatic GC patients [72]. In general, previously treated MSI-H tumors had higher RRs to pembrolizumab, nivolumab or the combination of nivolumab plus ipilimumab compared to non-MSI-H GC [36,37,38,40]. Additionally, MSI-H GC that had progressed after first-line therapy was more likely to respond to pembrolizumab versus paclitaxel (RR 46.7% vs. 16.7%) with better OS based on the phase 3 KEYNOTE-61 study but the difference was not statistically tested [38]. Therefore, ICIs may be more effective than chemotherapy for MSI-H GC that had progressed on prior chemotherapy. 

In the first-line setting, pembrolizumab with or without chemotherapy was compared with chemotherapy alone in HER2-negative, PD-L1 CPS ≥ 1 GC in the randomized, phase 3 KEYNOTE-62 study [39]. For those with CPS ≥10 irrespective of the MSI status, pembrolizumab improved median OS (mOS) versus chemotherapy (17.4 vs. 10.8 months, HR 0.69, 95% CI 0.49–0.97), but the difference was not statistically tested; pembrolizumab plus chemotherapy was not superior to chemotherapy (mOS 12.3 vs. 10.8 months, HR 0.85, 95% CI 0.62–1.17, *p* = 0.16) [39]. In the exploratory analysis of MSI-H tumors, pembrolizumab monotherapy prolonged mOS compared to chemotherapy in those with CPS ≥ 10 (NR vs. 13.6 months, HR 0.21, 95% CI 0.06–0.83). For CPS ≥ 1, pembrolizumab with or without chemotherapy were not superior to chemotherapy alone (mOS 12.5/10.6 vs. 11.1 months), but pembrolizumab monotherapy improved mOS compared to chemotherapy in those with MSI-H tumors (NR vs. 8.5 months, HR 0.29, 95% CI 0.11–0.81) [39]. ICIs are an emerging first-line therapy for MSI-H GC. Whether concurrent PD-L1 expression should also be a prerequisite for its first-line use needs to be investigated.

ICIs in combination with chemotherapy may soon become front-line therapy for metastatic GC. The addition of nivolumab to chemotherapy improved mOS compared to chemotherapy alone (14.4 vs. 11.1 months, HR 0.71, 95% CI 0.59–0.86, *p* < 0.0001) in treatment-naive, CPS ≥ 5 GC based on interval analysis of the CheckMate649 study [43]. However, the ATTRACTION-4 study in Asian patients did not demonstrate OS benefits of the immunochemotherapy approach, but this cohort was heavily treated with multiple lines of therapies [42]. Subgroup analysis with MSI-H GC may shed light on the role of ICIs as a first-line treatment for this population. In the perioperative setting, ICIs, in combination with chemotherapy and radiation therapy, are being investigated in patients with surgically resectable GC [91].

Overall, ICIs have made significant strides in the treatment of GC. Their success likely stems from a hot tumor microenvironment (TME) marked by MSI-H-, TMB-H- and PD-L1-positive tumors and pathological associations with Helicobacter pylori and EBV infections. In addition to chemotherapy, combining ICIs with targeted therapies such as inhibitors against the VEGF or HER-2 pathway that are capable of modulating the TME have produced encouraging results [92,93]. Sequential therapy from ICIs to chemotherapy is another promising strategy [94]. Subgroup analysis of MSI-H GC in these clinical studies will provide unique insights to further understanding of TME and resistance mechanisms to immunotherapy. 

## 4. Esophageal Squamous Cell Carcinoma and Adenocarcinoma

Esophageal cancer is histologically categorized into squamous cell carcinoma (ESCC) and adenocarcinoma (EAC). Siewert type I and II GEJ adenocarcinoma is commonly considered as esophageal cancer but clear demarcation between esophageal and gastric cancer remains controversial [79]. A comprehensive molecular analysis of esophageal tumors showed that ESCC and EAC have different genetic signatures [91]. Frequent amplifications of CCND1, SOX2 and TP65 were found in ESCC while ERBB2, VEGFA, GATA4 and GATA6 were more common in EAC [95]. 

The prevalence of MSI-H in esophageal cancer was reported ranging from 0–3.3% [10,28,29,44,67,68]. Some studies note that the presence of MSI-H was limited to EAC of GEJ origin (primarily Siewert type II and III disease), supporting that the rates of MSI-H tumors are much lower in esophageal cancer than gastric cancer [95,96]. In the KEYNOTE-180 study, one of 98 evaluable patients was found to have MSI-H esophageal tumor but did not respond to pembrolizumab [44]. Currently, there is insufficient evidence to conclude the role of MSI status in esophageal cancer. 

The percentage of TMB-H tumors seems higher in ESCC than EAC [67] and ESCC is more sensitive to ICIs compared to EAC (RR 14.3% vs. 5.2%) based on the KEYNOTE-180 study [44]. This was further supported by the phase 3 KEYNOTE-181 study, which showed that pembrolizumab versus chemotherapy as a second-line therapy for refractory esophageal cancer improved survival in patients with ESCC (mOS 8.2 vs. 7.1 months, HR 0.78, 95% CI 0.63–0.96, *p* = 0.0095) and those with CPS ≥ 10 irrespective of histology (mOS 9.3 vs. 6.7 months, HR 0.69, CI 95% 0.52–0.93, *p* = 0.0074) [45]. For previously treated ESCC, nivolumab also improved survival compared to chemotherapy (mOS 10.9 vs. 8.4 months, HR 0.77, 95% CI 0.62–0.96, *p* = 0.019), but the RR was lower (RR 19% vs. 22%) in the phase 3 ATTRACTION-3 study, which predominantly enrolled Asian patients [48]. No relevant interaction was observed in the pre-specified subgroup analysis by the levels of PD-L1 expression (cutoff points at 1, 5 and 10) in this study [48]. Taken together, ICIs are reasonable options for ESCC and PD-L1 CPS ≥ 10 esophageal cancer that have progressed on prior therapies. 

It is anticipated that ICIs will also become part of first-line therapy for metastatic ESCC and PD-L1 CPS ≥ 10 esophageal cancer. Interval analysis of the phase 3 KEYNOTE-590 study showed that pembrolizumab in combination with chemotherapy prolonged survival compared to chemotherapy alone in patients with ESCC (mOS 12.6 vs. 9.8 months, HR 0.72, 95% CI 0.60–0.88, *p* = 0.0006), CPS ≥ 10 (mOS 13.5 vs. 9.4 months, HR 0.62, 95% CI 0.49–0.78, *p* < 0.0001) and all esophageal cancer patients (mOS 12.4 vs. 9.8 months, HR 0.73, 95% CI 0.62–0.86, *p* <0.0001); similar benefits were also seen in PFS and RR [46]. Compared to non-MSI-H tumors, MSI-H esophageal cancer is likely to derive greater benefits from ICIs, but it remains unclear whether the combination of ICI plus chemotherapy is superior to ICI monotherapy for this group of patients. 

## 5. Small Bowel Adenocarcinoma

Small bowel cancer accounts for less than 5% of GI cancers with adenocarcinoma being the most common (~40%) histology [97,98]. It is a rare cancer despite that the small intestine makes up 75% of the GI tract. Small bowel adenocarcinoma (SBA) carries a worse prognosis than CRC (5-year OS rates, 34.9% vs. 51.5%, *p* < 0.0001) [99]. Although Lynch syndrome is a known risk factor for both SBA and CRC, there is no consensus on appropriate screening for SBA in this high-risk population yet [100]. 

Clinical management of SBA has been based on experience extrapolated from CRC but SBA is increasingly recognized as a distinct category of luminal cancers given improved understanding of its genetic landscape. Compared to CRC, rates of genomic alterations in SBA are higher in HER2 (9.5% vs. 5.1%, *p* = 0.001) and CDKN2A (14.5% vs. 2.6%, *p* < 0.001), lower in APC (26.8% vs. 75.9%, *p* < 0.001) and similar in BRAF (9.1% vs. 7.6%, *p* = 0.37) [101]. The duodenum is the most common site of SBA and the genetic profiles of duodenal SBA versus SBA of unspecified locations appear similar [98,101]. 

The prevalence of MSI-H and TMB-H tumors in SBA was, respectively, reported to be 2–8.3% and 8.3–10.2% [10,67,101]. One study containing 317 patients with SBA noted that all MSI-H tumors had intermediate (10–20 mut/Mb) to high (>20 mut/Mb) TMB [101]. MSI-H is a favorable prognostic biomarker in stage II and III CRC [102,103], and it may predict a lack of benefits from fluoropyrimidine-based adjuvant chemotherapy in stage II disease [104]. In SBA, MSI-H was found to be associated with early-stage disease and lower rates of recurrence in a retrospective study of 74 patients [105]. Additionally, dMMR/MSI-H was associated with improved cancer-specific survival in stage II SBA [106], improved postoperative cancer-specific survival and higher CPS, regardless of disease stage [107]. 

Although the predictive value of MSI status in SBA is not fully elucidated, MSI-H SBA is likely to respond to ICIs. Eight of 19 patients (42.1%) with previously treated MSI-H SBA in the KEYNOTE-158 study responded to pembrolizumab [35]. In a single-arm phase 2 study involving 40 patients with SBA refractory to at least one prior line of systemic therapy, three patients had confirmed response to pembrolizumab and two of them were MSI-H [49]. In addition, responders to pembrolizumab had lower Bim (a pro-apoptotic molecule) levels in circulating CD8+ T cells at baseline; upregulation of CX3CR1/granzyme B in these T cells was associated with improved survival [108]. Currently, fluorouracil-based chemotherapy remains the standard first-line treatment while ICIs are recommended as a second-line therapy for MSI-H SBA [109]. 

## 6. Anal Carcinoma

Anal carcinoma (AC) is primarily of squamous cell histology with human papillomavirus (HPV) infection detected in ~90% of AC [110,111]. Both HPV positivity and increased TILs were associated with improved survival and treatment responses to chemoradiation in AC [112,113,114,115], indicating that HPV infection may trigger immune response and enhance the activity of ICIs, similar to what has been observed in clinical trials with ICIs in squamous cell carcinoma of the head and neck [116,117]. 

MSI-H is rare in AC but TMB-H tumors constitute 8.3–33% of AC [34,67]. Regardless of the MSI status, ICIs targeting PD-1 are the preferred therapy for anal squamous cell carcinoma after disease progression on first-line chemotherapy [50,74,75,76]. A combined analysis of the KEYNOTE-28 and KEYNOTE-158 study reported an RR of 10.9% in 137 patients with higher RRs in the PD-L1 positive group versus negative group (14.0% vs. 3.3%); median DOR was not reached after a median follow-up of 11.7 months [75]. Nivolumab led to radiographic responses in 24% of 37 patients with a median DOR of 5.8 months; responders had higher expression of PD-L1 on tumors cells, PD-1, LAG-3 and TIM-3 in CD8+ T cells at baseline [50].

## 7. Hepatocellular Carcinoma

Incidence and mortality rates of hepatocellular carcinoma (HCC) are climbing [118], with viral hepatitis, alcoholic cirrhosis and nonalcoholic fatty liver disease/nonalcoholic steatohepatitis as known risk factors [119,120,121], emphasizing the importance of disease prevention and screening in those at risk. For patients with limited disease burden, partial hepatectomy and locoregional therapies are promising [122,123,124,125,126]. Liver transplantation is a curative treatment reserved for early-stage HCC only [127,128].

Historically, first-line systemic therapies inhibiting the VEGF pathway such as sorafenib and lenvatinib have limited efficacy in HCC, prolonging OS by 2–3 months [129,130]. With better understanding of chronic inflammation as a major mediator of tumorigenesis in HCC [131], immunotherapy has become an area of active research. Nivolumab with or without ipilimumab were shown to have significant activity in Child-Pugh A-B HCC that was previously treated with sorafenib based on the phase 2 CheckMate040 study [53,54,55]. Pembrolizumab also improved OS compared to placebo (mOS 13.9 vs. 10.6 months, HR 0.781, 95% CI 0.611–0.998, *p* = 0.0238) for Child-Pugh A HCC that was previously treated with sorafenib with an RR of 18.3% in the phase 3 KEYNOTE-240 study; patients with baseline α-fetoprotein <200 ng/mL or history of hepatitis B derived more survival benefits [51,52]. ICIs targeting PD-1 are, therefore, reasonable choices for HCC refractory to sorafenib with potentially longer OS than other approved agents including regorafenib [132], cabozantinib [133] and ramucirumab [134].

The combination of atezolizumab (ICI targeting PD-L1) plus bevacizumab as a first-line therapy for HCC is a major breakthrough. Compared to sorafenib, this combination improved RR (27.3% vs. 11.9%, *p* < 0.001) and OS (HR for death 0.58, 95% CI 0.42–0.79, *p* < 0.001) for Child-Pugh A HCC based on the phase 3 IMbrave150 study [57]. Nivolumab monotherapy, however, is not superior to sorafenib as a first-line therapy (mOS 16.4 vs. 14.7 months, HR 0.85, 95% CI 0.72–1.02, *p* = 0.0752; RR 15% vs. 7%) based on the phase 3 CheckMate459 study [56].

MSI-H occurs in less than 3% of HCC and MSI tends to be higher in patients with cirrhosis [28,29,67,69]. PD-L1 positive HCC was also shown to have higher RRs to ICIs [51,56]. Overall, further studies will need to be performed to interrogate the predictive value of MSI and PD-L1 in HCC. 

## 8. Biliary Tract Cancer

Biliary tract cancer (BTC) encompasses gallbladder cancer and cholangiocarcinoma, which can be further divided into intrahepatic and extrahepatic cholangiocarcinoma. BTC is often diagnosed at an advanced stage. Cisplatin plus gemcitabine is the standard first-line therapy for unresectable disease (RR of 26%, mOS 11.7 months) [135] and fluorouracil plus oxaliplatin for subsequent treatment (RR not reported, mOS 6.2 months) [136]. Tyrosine kinase inhibitors targeting NTRK, IDH1 and FGFR2 are efficacious in BTC with corresponding genetic alterations [137,138,139,140]. Due to limited therapeutic choice, BTC carries an extremely poor prognosis.

The prevalence of MSI-H BTC is less than 3% [10,29,30,67], but ICIs targeting PD-1 was shown to have significant antitumor activity in these tumors. In the KEYNOTE-158 study, 40.9% of 22 patients with MSI-H BTC that had progressed on prior therapy responded to pembrolizumab with a median OS of 24.3 months [35]. Given high RRs and prolonged OS, pembrolizumab is a reasonable alternative for patients with MSI-H BTC who are not candidates for chemotherapy. 

ICIs also have good activity in BTC refractory to prior therapy. A phase 2 study with nivolumab showed an RR of 11% (22% by investigator-assessed response; median DOR was NR after a median follow-up of 12.4 months) in previously treated BTC, and surprisingly, all responders had MMR proficient (pMMR) tumors [58]. PD-L1 expression in tumor specimens was statistically associated with prolonged PFS [58]. Adding ipilimumab to nivolumab further enhanced RRs (23%) in a phase 2 study, and again, none of the responders had MSI-H tumors [59]. A combined analysis of the KEYNOTE-28 and KEYNOTE-158 study reported an RR of 7.1% in 127 BTC patients with higher RRs in the PD-L1 positive group versus negative group [77]. Altogether, these data suggest that judicious selection of patients with BTC for ICIs will need to be guided by predictive biomarkers and warrants further studies.

## 9. Pancreatic Ductal Adenocarcinoma

Pancreatic ductal adenocarcinoma (PDAC) accounts for 90% of pancreatic cancer and chemotherapy currently remains the standard of care. Targeted therapies against the most common somatic mutations in PDAC, including KRAS, TP53, SMAD4 and CDKN2A, are being investigated with limited success [141]. Their therapeutic efficacy is likely impaired by desmoplasia, which consists of dense fibrosis produced by pancreatic stellate cells, posing significant barriers to drug penetration [142,143].

The rates of MSI-H and TMB-H tumors are low in PDAC [10,29,30,67,71]. In general, PDAC also appears much more resistant to ICIs than other GI tumors. Only 18.2% of 22 patients with previously treated MSI-H PDAC responded to pembrolizumab in the KEYNOTE-158 study [35]. Ipilimumab monotherapy did not result in any radiographic responses [78]. Durvalumab with or without tremelimumab led to responses in 3.1% and 0% of patients with refractory PDAC [60]. One of the three responders was MSI-H with germline dMMR [60]. The resistance of PDAC to ICIs has been attributed to a TME devoid of infiltrating immune cells [144]. It was suggested that ICIs alone are not likely to induce a tumor response in PDAC, and future studies should focus on developing strategies to prime the TME to enhance the reactions of ICIs [145].

## 10. Tumors of the Ampulla of Vater

Tumors arising in the vicinity of the ampulla of Vater are rare and occur more frequently in patients with Lynch syndrome. They have a better prognosis than periampullary cancers such as BTC and PDAC. Fluoropyrimidine- or gemcitabine-based chemotherapy are often recommended for advanced ampullary cancers that are not amenable to surgical resection, but as of yet, there is no consensus on systemic therapy due to the lack of randomized controlled trials [146]. Interestingly, MSI-H/dMMR was found in up to 18% of ampullary cancers and was associated with better survival [147,148], indicating a potential role of ICIs in the management of this malignancy.

## 11. GI Neuroendocrine Neoplasms

Neuroendocrine neoplasms of the GI origin comprise a diverse group of malignancies. In general, they are categorized into neuroendocrine carcinomas (NECs) and neuroendocrine tumors (NETs). NECs are distinct from NETs as NECs are poorly differentiated with a poor prognosis [149]. NETs are further divided into high, intermediate and low grade based on the mitotic rate and Ki-67 index according to the 2019 World Health Organization guidelines [149]. Classification of GI NETs has been evolving over the past decade, but pancreatic NETs (PNETs) remain a unique category with potentially worse prognosis but better responses to chemotherapy [150]. 

Although MSI-H was reported to be 0% in PNETs [67], 3.6% in NETs [10] and 12.4% in NECs [151], ICIs may be effective in high-grade NETs and NECs based on preliminary results from a few clinical trials [152]. The phase 2 DART SWOG 1609 study aims to evaluate the efficacy of the nivolumab plus ipilimumab in patients with rare tumors and reported an RR of 13% (2/15) in patients with previously treated, non-pancreatic GI NETs [61]; the PNET cohort is accruing. Both responders had high-grade NETs and MSI data were not available [61]. The overall RR was 25% for nonpancreatic NETs and higher in high-grade NETs versus intermediate/low-grade NETs (44% vs. 0%, *p* = 0.004) [61]. Another phase 2 study that evaluated the antitumor activity of spartalizumab, a humanized PD-1 antibody under investigation, led to an RR of 7.4% and 4.8% in NETs and NECs, respectively [153].

The efficacy of pembrolizumab monotherapy has also been evaluated. The KEYNOTE-158 study showed an RR of 3.7% in 107 patients with previously treated, well to moderately differentiated NETs [80,154]. All four responders had GI NETs (three PNETs and one rectal NET) and none of them was positive for PD-L1 expression [154]. The KEYNOTE-28 study recruited PD-L1 positive tumors only and showed an RR of 12% and 6.3% in 25 and 16 patients, respectively, with carcinoid tumors and PNETs refractory to prior therapy [79]. Furthermore, the combination of atezolizumab plus bevacizumab resulted in an RR of 20% in the PNET cohort and 15% in the extrapancreatic NET cohort [62]. Similar to PDAC, ICIs are likely more efficacious in GI NETs or NECs when used in combination with agents that modulate the TME to augment the activity of immune cells. 

## 12. Future Directions

### 12.1. Overcoming Resistance to ICIs

Although MSI-H predicts favorable treatment response to ICIs, RRs vary depending on tumor type and treatment responses are not exclusively seen in MSI-H tumors, raising two important questions. First, are there tumor-specific characteristics that we could harness to improve therapeutic efficacy of ICIs and develop novel strategies to target treatment? Second, are there patient-specific factors that we need to overcome on the individual level?

Schreiber et al. proposed the concept of cancer immunoediting in 2011, illustrating that the TME is capable of suppressing and promoting tumor development depending on cellular signaling [155]. Subsequently, O’Donnell et al. divided TME into four types based on TMB (high vs. low) and inflammation gene signatures (high vs. low), taking into consideration both intrinsic tumor characteristics and immune reactions in response to tumor antigens [144]. According to this framework, tumors with high TMB and inflammatory TME such as gastric cancer are likely to respond to ICIs because tumor antigens are presented to a milieu with abundant immune cells. However, ICIs targeting PD-1 could also exert an off-target effect on PD-1 positive tumor-associated macrophages (TAMs) and dampen the adaptive immune response [156]. Therefore, focusing on mechanisms that reduce inhibitory signaling to the adaptive immune system will be critical to improve the use of ICIs in this type of tumors. On the other hand, tumors with low TMB and lack of TILs such as pancreatic cancer will be resistant to ICIs. Promoting antigen presentation and immune cell infiltration will be the first step to enable the action of ICIs. Altogether, developing strategies to overcome resistance to ICIs should be ideally guided by the nature of the tumor and TME.

On the individual level, patients could have primary resistance (no clinical response or disease control after initial exposure to treatment) or acquired resistance (cancer progresses after an initial response or disease control on treatment) to ICIs. Many of these pathways overlap [157,158] with altered interferon-γ signaling as a result of JAK1/2 mutation being a classical example [159,160]. Primary resistance to ICIs can be partly explained by intratumoral and intertumoral heterogeneity of infiltrating immune cells. Yoon et al. reported that in a retrospective study of patients with CRC, the density of TILs was higher at the invasive margin than the tumor core within a dMMR tumor (i.e., intratumoral heterogeneity) and the variance of TIL densities were much higher in dMMR tumors compared to pMMR tumors (i.e., intertumoral heterogeneity) [161], suggesting that dMMR tumors have different distribution of TILs, which may lead to differential responses to ICIs. 

Acquired resistance to ICIs may arise from mutations in certain genes. Le et al. noted five cases of acquired resistance after initial response to pembrolizumab and performed exome sequencing of both primary and metastatic tumors from two of these patients [24]. The mutation profile of metastatic lesions resembles that of primary lesions in each patient, but a new/second mutation in beta-2 microglobulin (B2M) gene was identified in the metastatic tumor of respective patients [24], suggesting that B2M mutations may contribute to secondary resistance to ICIs. As more mechanisms of resistance are being revealed, we may be able to develop molecular therapies that target these pathways in the future.

To date, active research is ongoing to test combination therapies with ICIs, novel combinations of ICIs, sequential therapies with chemotherapy, radiation therapy and ICIs, cellular therapies, oncolytic virus and microbiota modulation with ICIs. Fundamental rationales for these studies all build upon understanding of the response and resistance mechanisms to immunotherapy. Conducting well-designed mechanistic studies along with these clinical trials will, thus, be crucial to sustain the momentum of immuno-oncology as a field.

### 12.2. Perioperative Use of ICIs

Extending the use of ICIs to the perioperative setting is also an exciting frontier [162]. The CheckMate577 study showed that adjuvant nivolumab improved DFS compared to placebo (median 22.4 months vs. 11.0 months, HR 0.69, 95% CI 0.56–0.86, *p* = 0.0003) in patients with esophageal cancer following neoadjuvant chemoradiation and surgical resection [163], providing first-hand evidence to support that ICIs may benefit cancer patients with early-stage disease. Demonstrating the clinical efficacy of ICIs in the neoadjuvant setting is challenging, as radiation therapy and surgical resection are cytoreductive in nature and perioperative serial radiographic measurements do not solely reflect tumor response to systemic treatment. Careful grading of pathological responses, serial metabolic imaging and circulating tumor DNA (ctDNA) could be potential solutions, but prospective studies need to be performed to validate their clinical utility.

### 12.3. Atypical Responses to ICIs

Another interesting phenomenon that deserves more attention is whether MSI-H tumors are associated with higher rates of atypical responses to ICIs (e.g., pseudoprogression and hyperprogression) as their TME is substantially enriched with immune cells and cytokines compared to non-MSI-H tumors. Atypical responses to ICIs were reported in up to 29% of patients based on existing criteria that is not yet standardized [164,165,166,167,168] and they could lead to either premature or delayed discontinuation of ICIs, undermining patient care. Currently, we do not have any tools that can reliably differentiate atypical treatment responses. Studies that evaluate the use of molecular signatures such as ctDNA in disease monitoring will potentially expedite the discovery of novel biomarkers that can directly measure host immunity against tumor cells, providing rationales to improve the clinical management of MSI-H tumors. 

## 13. Conclusions

The approval of ICIs for the treatment of MSI-H tumors represents a paradigm shift in oncology. Since ICIs have become part of front-line therapies for unresectable or metastatic solid tumors, we are in the search of universal predictive biomarkers that consistently identify proper candidates for immunotherapy across tumor types. MSI, TMB and PD-L1 have been widely investigated and proven useful in GI cancers. Studies have also shown that favorable clinical outcomes to immunotherapy were associated with subsets of TILs, T cell receptor diversity, cytokine levels, metabolites in the peripheral blood etc. in certain tumor groups [169,170,171,172,173,174,175,176]. As novel precision medicine technologies are being developed, we envision that therapeutic decisions in the near future will be guided by a wealth of genetic information and composite biomarkers in addition to the MSI status. 

## Figures and Tables

**Table 1:** Major classes of immune checkpoint inhibitors indicated for gastrointestinal cancers.

Target	Generic Name	Disease Category	FDA Indications			
			Required Biomarker	Required Prior Therapy	Monotherapy ^1^	Combination ^1^
PD-1	Nivolumab	CRC	MSI-H/dMMR	Fluoropyrimidine, oxaliplatin and irinotecan	**√**	With/without ipilimumab
		HCC	None	Sorafenib	**√**	With/without ipilimumab
PD-1	Pembrolizumab	Solid tumor	MSI-H/dMMR or TMB-H ^2^	Previously treated with no satisfactory alternative treatment options	**√**	×
		CRC	MSI-H/dMMR	None or fluoropyrimidine, oxaliplatin and irinotecan	**√**	×
		HCC	None	Sorafenib	**√**	×
		GC	PD-L1 CPS ≥ 1	At least two prior lines of therapy including fluoropyrimidine- and platinum-containing chemotherapy and if appropriate, HER2 targeted therapy	**√**	×
PD-L1	Atezolizumab	HCC	None	None	×	With bevacizumab
CTLA-4	Ipilimumab	CRC	MSI-H/dMMR	Fluoropyrimidine, oxaliplatin and irinotecan	×	With nivolumab
		HCC	None	Sorafenib	×	With nivolumab

Abbreviations: CRC, colorectal cancer; HCC, hepatocellular carcinoma; GC, gastric cancer; MSI-H, microsatellite instability-high; dMMR, deficient DNA mismatch repair; TMB, tumor mutational burden. ^1^ **√** indicates FDA approval of therapy and **×** indicates lack of FDA approval. ^2^ TMB-H is defined as TMB greater or equal to 10 mutations/megabases.

**Table 2:** Major clinical trials involving immune checkpoint inhibitors in non-colorectal gastrointestinal cancers.

Disease Category	Clinical Study (Phase)	References	Interventions	Inclusion Criteria	Major Findings Pertinent to MSI
Solid tumor	KEYNOTE-28 (1b)	[33]	Single-arm P	PD-L1 positive, previously treated	Higher RR in MSI-H tumors vs. non-MSI-H tumors
	KEYNOTE-158 (2)	[34,35]	Single-arm P	Previously treated	Higher RR in MSI-H or TMB-H tumors vs. non-MSI-H tumors
GC	KEYNOTE-12 GC cohort (1b)	[36]	Single-arm P	PD-L1 positive, previously treated	Higher RR in MSI-H tumors vs. non-MSI-H tumors
	KEYNOTE-59 (2)	[37]	Single-arm P	Progression after ≥2 prior lines of therapy including platinum and fluoropyrimidine	Improved RR and survival in MSI-H tumors vs. non-MSI-H tumors
	KEYNOTE-61 (3)	[38]	P vs. paclitaxel	Progression after first-line therapy with platinum and fluoropyrimidine	P improved RR and survival vs. paclitaxel in MSI-H tumors
	KEYNOTE-62 (3)	[39]	P vs. P + C vs. C	CPS ≥1, HER2 negative and treatment-naive	P improved RR and survival vs. C alone in MSI-H tumors
	CheckMate032 (1/2)	[40]	N vs. N + I	Previously treated	Higher RRs in MSI-H tumors vs. non-MSI-H tumors
	ATTRACTION-2 (3)	[41]	N vs. placebo	Previously treated with ≥2 prior lines of therapy	N/A
	ATTRACTION-4 (2/3)	[42]	N + C vs. placebo + C	HER2 negative, treatment-naive	N/A
	CheckMate649 (3)	[43]	N + C vs. N + I vs. C	HER2 negative, treatment-naive	N/A
EAC and ESCC	KEYNOTE-180 (2)	[44]	Single-arm P	Progression after ≥2 prior lines of therapy	Only one patient had an MSI-H tumor but did not respond to P
	KEYNOTE-181 (3)	[45]	P vs. C	Progression after first-line therapy	NA
	KEYNOTE-590 (3)	[46]	P + C vs. placebo + C	Treatment-naive	NA
ESCC	ATTRACTION-1 (2)	[47]	Single-arm N	Refractory or intolerant to fluoropyrimidine-, platinum- or taxane-based C	NA
	ATTRACTION-3 (3)	[48]	N vs. C	Refractory or intolerant to first-line therapy with platinum and fluoropyrimidine	NA
SBA	ZEBRA (2)	[49]	Single-arm P	Previously treated	Higher RR in MSI-H tumors vs. non-MSI-H tumors
AC	NCI9673 (2)	[50]	N vs. N + I	Previously treated	NA
HCC	KEYNOTE-224 (2)	[51]	Single-arm P	Refractory or intolerant to sorafenib	NA
	KEYNOTE-240 (3)	[52]	P vs. placebo	Refractory or intolerant to first-line therapy with sorafenib	NA
	CheckMate040 (1/2)	[53,54,55]	N vs. N + I	Previously treated with sorafenib	NA
	CheckMate459 (3)	[56]	N vs. sorafenib	Treatment-naive	NA
	IMbrave150 (3)	[57]	A + B vs. sorafenib	Treatment-naive	NA
BTC	NCT02829918 (2)	[58]	Single-arm N	Progression after 1–3 prior lines of therapy	All responders had pMMR tumors
	NCT02923934 (2)	[59]	Single-arm N + I	Prior therapy allowed	All responders had MSS/MSI-L tumors
PDAC	NCT02558894 (2)	[60]	D vs. D + T	Progression after first-line therapy with fluorouracil or gemcitabine	One out of three responders had MSI-H tumor with germline dMMR
GI NET	DART SWOG 1609 (2)	[61]	Single-arm N + I	Progression after ≥1 prior line of therapy	NA
	NCT03074513 (2)	[62]	Single-arm A + B	Prior therapy allowed	NA

Abbreviations: disease category—GC, gastric cancer; EAC, esophageal adenocarcinoma; ESCC, esophageal squamous cell carcinoma; SBA, small bowel adenocarcinoma; AC, anal cancer; HCC, hepatocellular carcinoma; BTC, biliary tract cancer; PDAC, pancreatic adenocarcinoma; GI NET, gastrointestinal neuroendocrine tumor; interventions—P, pembrolizumab; C, chemotherapy; N, nivolumab; I, ipilimumab; A, atezolizumab; B, bevacizumab; D, durvalumab; T, tremelimumab; results—RR, response rate; NA, not available.

**Table 3:** Prevalence of MSI-H, TMB-H and PD-L1 in non-colorectal gastrointestinal cancers and response rates to immune checkpoint inhibitors (ICIs).

Tumor Type	Biomarker (%) [10,28,29,30,34,67,68,69,70,71]			Response Rates to ICI Monotherapy (%)		
	MSI-H	TMB	PD-L1	First Line ^1^	Beyond First Line ^1^	MSI-H Tumors ^2^
Gastric cancer	4–25	3.1–13	6.6–30.7	14.8 [39]	11.6–22 [36,37,38]	45.8–85.7 [35,36,72]
Esophageal cancer	0–3.3	0.5–17	16.2–24.9	Not reported [46]	9.9–30 [44,45,73]	Not reported [35]
Small bowel adenocarcinoma	2–8.3	8.3–10.2	10.5–16.7	-	7.5 [49]	42.1 [35]
Anal cancer	0	8.3–33	38	-	10.9–24 [50,74,75,76]	-
Hepatocellular carcinoma	0–2.9	1.4–7	6.1–9.6	15 [56]	10.2–20 [51,52,53,54]	-
Biliary tract cancer	1.4–2.5	3.4–26	8.5–18.6	-	7.1–11 [58,77]	40.9 [35]
Pancreatic adenocarcinoma	0–5.3	1.2–1.4	8.6–21.6	0 [78]	0 [60]	18.2 [35]
Pancreatic neuroendocrine tumor	0	1.3	2.9	-	6.3–7.5 [79,80]	-

^1^ Both MSI-H and MSS/MSI-L tumors were included. ^2^ Response rates in MSI-H tumors irrespective of lines of therapy.

## References

[1] Ellegren H. (2004). Microsatellites: Simple sequences with complex evolution. Nat. Rev. Genet..

[2] Kane M.F., Loda M., Gaida G.M., Lipman J., Mishra R., Goldman H., Jessup J.M., Kolodner R. (1997). Methylation of the hMLH1 promoter correlates with lack of expression of hMLH1 in sporadic colon tumors and mismatch repair-defective human tumor cell lines. Cancer Res..

[3] Ligtenberg M.J., Kuiper R.P., Chan T.L., Goossens M., Hebeda K.M., Voorendt M., Lee T.Y., Bodmer D., Hoenselaar E., Hendriks-Cornelissen S.J. (2009). Heritable somatic methylation and inactivation of MSH2 in families with Lynch syndrome due to deletion of the 3′ exons of TACSTD1. Nat. Genet..

[4] Lynch H.T., de la Chapelle A. (2003). Hereditary colorectal cancer. N. Engl. J. Med..

[5] Lindor N.M., Burgart L.J., Leontovich O., Goldberg R.M., Cunningham J.M., Sargent D.J., Walsh-Vockley C., Petersen G.M., Walsh M.D., Leggett B.A. (2002). Immunohistochemistry Versus Microsatellite Instability Testing in Phenotyping Colorectal Tumors. J. Clin. Oncol..

[6] Boland C.R., Thibodeau S.N., Hamilton S.R., Sidransky D., Eshleman J.R., Burt R.W., Meltzer S.J., Rodriguez-Bigas M.A., Fodde R., Ranzani G.N. (1998). A National Cancer Institute Workshop on Microsatellite Instability for cancer detection and familial predisposition: Development of international criteria for the determination of microsatellite instability in colorectal cancer. Cancer Res..

[7] Hempelmann J.A., Lockwood C.M., Konnick E.Q., Schweizer M.T., Antonarakis E.S., Lotan T.L., Montgomery B., Nelson P.S., Klemfuss N., Salipante S.J. (2018). Microsatellite instability in prostate cancer by PCR or next-generation sequencing. J. Immunother. Cancer.

[8] Azad N.S., Gray R.J., Overman M.J., Schoenfeld J.D., Mitchell E.P., Zwiebel J.A., Sharon E., Streicher H., Li S., McShane L.M. (2020). Nivolumab Is Effective in Mismatch Repair-Deficient Noncolorectal Cancers: Results From Arm Z1D-A Subprotocol of the NCI-MATCH (EAY131) Study. J. Clin. Oncol..

[9] Berardinelli G.N., Scapulatempo-Neto C., Durães R., Antônio de Oliveira M., Guimarães D., Reis R.M. (2018). Advantage of HSP110 (T17) marker inclusion for microsatellite instability (MSI) detection in colorectal cancer patients. Oncotarget.

[10] Vanderwalde A., Spetzler D., Xiao N., Gatalica Z., Marshall J. (2018). Microsatellite instability status determined by next-generation sequencing and compared with PD-L1 and tumor mutational burden in 11,348 patients. Cancer Med..

[11] Dudley J.C., Lin M.-T., Le D.T., Eshleman J.R. (2016). Microsatellite Instability as a Biomarker for PD-1 Blockade. Clin. Cancer Res..

[12] Wu Y., Berends M.J., Mensink R.G., Kempinga C., Sijmons R.H., van der Zee A.G., Hollema H., Kleibeuker J.H., Buys C.H., Hofstra R.M. (1999). Association of hereditary nonpolyposis colorectal cancer-related tumors displaying low microsatellite instability with MSH6 germline mutations. Am. J. Hum. Genet..

[13] Smyrk T.C., Watson P., Kaul K., Lynch H.T. (2001). Tumor-infiltrating lymphocytes are a marker for microsatellite instability in colorectal carcinoma. Cancer.

[14] Jenkins M.K., Taylor P.S., Norton S.D., Urdahl K.B. (1991). CD28 delivers a costimulatory signal involved in antigen-specific IL-2 production by human T cells. J. Immunol..

[15] Dong H., Zhu G., Tamada K., Chen L. (1999). B7-H1, a third member of the B7 family, co-stimulates T-cell proliferation and interleukin-10 secretion. Nat. Med..

[16] Ishida Y., Agata Y., Shibahara K., Honjo T. (1992). Induced expression of PD-1, a novel member of the immunoglobulin gene superfamily, upon programmed cell death. EMBO J..

[17] Krummel M.F., Allison J.P. (1995). CD28 and CTLA-4 have opposing effects on the response of T cells to stimulation. J. Exp. Med..

[18] Brahmer J.R., Drake C.G., Wollner I., Powderly J.D., Picus J., Sharfman W.H., Stankevich E., Pons A., Salay T.M., McMiller T.L. (2010). Phase I study of single-agent anti-programmed death-1 (MDX-1106) in refractory solid tumors: Safety, clinical activity, pharmacodynamics, and immunologic correlates. J. Clin. Oncol..

[19] Topalian S.L., Hodi F.S., Brahmer J.R., Gettinger S.N., Smith D.C., McDermott D.F., Powderly J.D., Carvajal R.D., Sosman J.A., Atkins M.B. (2012). Safety, activity, and immune correlates of anti-PD-1 antibody in cancer. N. Engl. J. Med..

[20] Lipson E.J., Sharfman W.H., Drake C.G., Wollner I., Taube J.M., Anders R.A., Xu H., Yao S., Pons A., Chen L. (2013). Durable cancer regression off-treatment and effective reinduction therapy with an anti-PD-1 antibody. Clin. Cancer Res..

[21] Rizvi N.A., Hellmann M.D., Snyder A., Kvistborg P., Makarov V., Havel J.J., Lee W., Yuan J., Wong P., Ho T.S. (2015). Mutational landscape determines sensitivity to PD-1 blockade in non–small cell lung cancer. Science.

[22] Van Allen E.M., Miao D., Schilling B., Shukla S.A., Blank C., Zimmer L., Sucker A., Hillen U., Geukes Foppen M.H., Goldinger S.M. (2015). Genomic correlates of response to CTLA-4 blockade in metastatic melanoma. Science.

[23] Yarchoan M., Hopkins A., Jaffee E.M. (2017). Tumor Mutational Burden and Response Rate to PD-1 Inhibition. N. Engl. J. Med..

[24] Le D.T., Durham J.N., Smith K.N., Wang H., Bartlett B.R., Aulakh L.K., Lu S., Kemberling H., Wilt C., Luber B.S. (2017). Mismatch repair deficiency predicts response of solid tumors to PD-1 blockade. Science.

[25] Overman M.J., McDermott R., Leach J.L., Lonardi S., Lenz H.J., Morse M.A., Desai J., Hill A., Axelson M., Moss R.A. (2017). Nivolumab in patients with metastatic DNA mismatch repair-deficient or microsatellite instability-high colorectal cancer (CheckMate 142): An open-label, multicentre, phase 2 study. Lancet Oncol..

[26] Overman M.J., Lonardi S., Wong K.Y.M., Lenz H.J., Gelsomino F., Aglietta M., Morse M.A., Van Cutsem E., McDermott R., Hill A. (2018). Durable Clinical Benefit With Nivolumab Plus Ipilimumab in DNA Mismatch Repair-Deficient/Microsatellite Instability-High Metastatic Colorectal Cancer. J. Clin. Oncol..

[27] Amonkar M., Lorenzi M., Zhang J., Mehta S., Liaw K.-L. (2019). Structured literature review (SLR) and meta-analyses of the prevalence of microsatellite instability high (MSI-H) and deficient mismatch repair (dMMR) in gastric, colorectal, and esophageal cancers. J. Clin. Oncol..

[28] Hause R.J., Pritchard C.C., Shendure J., Salipante S.J. (2016). Classification and characterization of microsatellite instability across 18 cancer types. Nat. Med..

[29] Bonneville R., Krook M.A., Kautto E.A., Miya J., Wing M.R., Chen H.-Z., Reeser J.W., Yu L., Roychowdhury S. (2017). Landscape of Microsatellite Instability Across 39 Cancer Types. JCO Precis. Oncol..

[30] Abrha A., Shukla N.D., Hodan R., Longacre T., Raghavan S., Pritchard C.C., Fisher G., Ford J., Haraldsdottir S. (2020). Universal Screening of Gastrointestinal Malignancies for Mismatch Repair Deficiency at Stanford. JNCI Cancer Spectr..

[31] Andre T., Shiu K.-K., Kim T.W., Jensen B.V., Jensen L.H., Punt C.J.A., Smith D.M., Garcia-Carbonero R., Benavides M., Gibbs P. (2020). Pembrolizumab versus chemotherapy for microsatellite instability-high/mismatch repair deficient metastatic colorectal cancer: The phase 3 KEYNOTE-177 Study. J. Clin. Oncol..

[32] André T., Shiu K.-K., Kim T.W., Jensen B.V., Jensen L.H., Punt C., Smith D., Garcia-Carbonero R., Benavides M., Gibbs P. (2020). Pembrolizumab in Microsatellite-Instability–High Advanced Colorectal Cancer. N. Engl. J. Med..

[33] O′Neil B.H., Wallmark J.M., Lorente D., Elez E., Raimbourg J., Gomez-Roca C., Ejadi S., Piha-Paul S.A., Stein M.N., Abdul Razak A.R. (2017). Safety and antitumor activity of the anti-PD-1 antibody pembrolizumab in patients with advanced colorectal carcinoma. PLoS ONE.

[34] Marabelle A., Fakih M., Lopez J., Shah M., Shapira-Frommer R., Nakagawa K., Chung H.C., Kindler H.L., Lopez-Martin J.A., Miller W.H. (2020). Association of tumour mutational burden with outcomes in patients with advanced solid tumours treated with pembrolizumab: Prospective biomarker analysis of the multicohort, open-label, phase 2 KEYNOTE-158 study. Lancet Oncol..

[35] Marabelle A., Le D.T., Ascierto P.A., Di Giacomo A.M., De Jesus-Acosta A., Delord J.P., Geva R., Gottfried M., Penel N., Hansen A.R. (2020). Efficacy of Pembrolizumab in Patients With Noncolorectal High Microsatellite Instability/Mismatch Repair-Deficient Cancer: Results From the Phase II KEYNOTE-158 Study. J. Clin. Oncol..

[36] Muro K., Chung H.C., Shankaran V., Geva R., Catenacci D., Gupta S., Eder J.P., Golan T., Le D.T., Burtness B. (2016). Pembrolizumab for patients with PD-L1-positive advanced gastric cancer (KEYNOTE-012): A multicentre, open-label, phase 1b trial. Lancet Oncol..

[37] Fuchs C.S., Doi T., Jang R.W., Muro K., Satoh T., Machado M., Sun W., Jalal S.I., Shah M.A., Metges J.P. (2018). Safety and Efficacy of Pembrolizumab Monotherapy in Patients With Previously Treated Advanced Gastric and Gastroesophageal Junction Cancer: Phase 2 Clinical KEYNOTE-059 Trial. JAMA Oncol..

[38] Shitara K., Özgüroğlu M., Bang Y.J., Di Bartolomeo M., Mandalà M., Ryu M.H., Fornaro L., Olesiński T., Caglevic C., Chung H.C. (2018). Pembrolizumab versus paclitaxel for previously treated, advanced gastric or gastro-oesophageal junction cancer (KEYNOTE-061): A randomised, open-label, controlled, phase 3 trial. Lancet.

[39] Shitara K., Van Cutsem E., Bang Y.J., Fuchs C., Wyrwicz L., Lee K.W., Kudaba I., Garrido M., Chung H.C., Lee J. (2020). Efficacy and Safety of Pembrolizumab or Pembrolizumab Plus Chemotherapy vs Chemotherapy Alone for Patients With First-line, Advanced Gastric Cancer: The KEYNOTE-062 Phase 3 Randomized Clinical Trial. JAMA Oncol..

[40] Janjigian Y.Y., Bendell J., Calvo E., Kim J.W., Ascierto P.A., Sharma P., Ott P.A., Peltola K., Jaeger D., Evans J. (2018). CheckMate-032 Study: Efficacy and Safety of Nivolumab and Nivolumab Plus Ipilimumab in Patients With Metastatic Esophagogastric Cancer. J. Clin. Oncol..

[41] Kang Y.-K., Boku N., Satoh T., Ryu M.-H., Chao Y., Kato K., Chung H.C., Chen J.-S., Muro K., Kang W.K. (2017). Nivolumab in patients with advanced gastric or gastro-oesophageal junction cancer refractory to, or intolerant of, at least two previous chemotherapy regimens (ONO-4538-12, ATTRACTION-2): A randomised, double-blind, placebo-controlled, phase 3 trial. Lancet.

[42] Boku N., Ryu M., Oh D.-Y., Oh S.C., Oh H.C., Chung K., Lee T., Omori K., Shitara S., Sakuramoto I.J. (2020). Nivolumab plus chemotherapy versus chemotherapy alone in patients with previously untreated advanced or recurrent gastric/gastroesophageal junction (G/GEJ) cancer: ATTRACTION-4 (ONO-4538-37) study. Ann. Oncol..

[43] Moehler M., Shitara K., Garrido P., Salman L., Shen L., Wyrwicz K., Yamaguchi T., Skoczylas A. (2020). Campos Bragagnoli, T.; Liu, M.; et al. Nivolumab (nivo) plus chemotherapy (chemo) versus chemo as first-line (1L) treatment for advanced gastric cancer/gastroesophageal junction cancer (GC/GEJC)/esophageal adenocarcinoma (EAC): First results of the CheckMate 649 study. Ann. Oncol..

[44] Shah M.A., Kojima T., Hochhauser D., Enzinger P., Raimbourg J., Hollebecque A., Lordick F., Kim S.-B., Tajika M., Kim H.T. (2019). Efficacy and Safety of Pembrolizumab for Heavily Pretreated Patients With Advanced, Metastatic Adenocarcinoma or Squamous Cell Carcinoma of the Esophagus: The Phase 2 KEYNOTE-180 Study. JAMA Oncol..

[45] Kojima T., Muro K., Francois E., Hsu C.-H., Moriwaki T., Kim S.-B., Lee S.-H., Bennouna J., Kato K., Lin S. (2019). Pembrolizumab versus chemotherapy as second-line therapy for advanced esophageal cancer: Phase III KEYNOTE-181 study. J. Clin. Oncol..

[46] Kato K., Sun J.M., Shah M.A., Enzinger P.C., Adenis A., Doi T., Kojima T., Metges J.P., Li Z., Kim S.B. (2020). LBA8_PR Pembrolizumab plus chemotherapy versus chemotherapy as first-line therapy in patients with advanced esophageal cancer: The phase 3 KEYNOTE-590 study. Ann. Oncol..

[47] Kudo T., Hamamoto Y., Kato K., Ura T., Kojima T., Tsushima T., Hironaka S., Hara H., Satoh T., Iwasa S. (2017). Nivolumab treatment for oesophageal squamous-cell carcinoma: An open-label, multicentre, phase 2 trial. Lancet Oncol..

[48] Kato K., Cho B.C., Takahashi M., Okada M., Lin C.Y., Chin K., Kadowaki S., Ahn M.J., Hamamoto Y., Doki Y. (2019). Nivolumab versus chemotherapy in patients with advanced oesophageal squamous cell carcinoma refractory or intolerant to previous chemotherapy (ATTRACTION-3): A multicentre, randomised, open-label, phase 3 trial. Lancet Oncol..

[49] Pedersen K., Foster N., Overman M., Boland P., Kim S., Arrambide K., Jaszewski B., Welch J., Wilson R., McWilliams R. (2019). ZEBRA: An ACCRU/IRCI multicenter phase 2 study of pembrolizumab in patients with advanced small bowel adenocarcinoma (SBA). Ann. Oncol..

[50] Morris V.K., Salem M.E., Nimeiri H., Iqbal S., Singh P., Ciombor K., Polite B., Deming D., Chan E., Wade J.L. (2017). Nivolumab for previously treated unresectable metastatic anal cancer (NCI9673): A multicentre, single-arm, phase 2 study. Lancet Oncol..

[51] Zhu A.X., Finn R.S., Edeline J., Cattan S., Ogasawara S., Palmer D., Verslype C., Zagonel V., Fartoux L., Vogel A. (2018). Pembrolizumab in patients with advanced hepatocellular carcinoma previously treated with sorafenib (KEYNOTE-224): A non-randomised, open-label phase 2 trial. Lancet Oncol..

[52] Finn R.S., Ryoo B.Y., Merle P., Kudo M., Bouattour M., Lim H.Y., Breder V., Edeline J., Chao Y., Ogasawara S. (2020). Pembrolizumab As Second-Line Therapy in Patients With Advanced Hepatocellular Carcinoma in KEYNOTE-240: A Randomized, Double-Blind, Phase III Trial. J. Clin. Oncol..

[53] El-Khoueiry A.B., Sangro B., Yau T., Crocenzi T.S., Kudo M., Hsu C., Kim T.Y., Choo S.P., Trojan J., Welling T.H.R. (2017). Nivolumab in patients with advanced hepatocellular carcinoma (CheckMate 040): An open-label, non-comparative, phase 1/2 dose escalation and expansion trial. Lancet.

[54] Kudo M., Matilla A., Santoro A., Melero I., Gracian A.C., Acosta-Rivera M., Choo S.P., El-Khoueiry A.B., Kuromatsu R., El-Rayes B.F. (2019). Checkmate-040: Nivolumab (NIVO) in patients (pts) with advanced hepatocellular carcinoma (aHCC) and Child-Pugh B (CPB) status. J. Clin. Oncol..

[55] Yau T., Kang Y.K., Kim T.Y., El-Khoueiry A.B., Santoro A., Sangro B., Melero I., Kudo M., Hou M.M., Matilla A. (2020). Efficacy and Safety of Nivolumab Plus Ipilimumab in Patients With Advanced Hepatocellular Carcinoma Previously Treated With Sorafenib: The CheckMate 040 Randomized Clinical Trial. JAMA Oncol..

[56] Yau T., Park J.W., Finn R.S., Cheng A.L., Mathurin P., Edeline J., Kudo M., Han K.H., Harding J.J., Merle P. (2019). CheckMate 459: A randomized, multi-center phase III study of nivolumab (NIVO) vs sorafenib (SOR) as first-line (1L) treatment in patients (pts) with advanced hepatocellular carcinoma (aHCC). Ann. Oncol..

[57] Finn R.S., Qin S., Ikeda M., Galle P.R., Ducreux M., Kim T.Y., Kudo M., Breder V., Merle P., Kaseb A.O. (2020). Atezolizumab plus Bevacizumab in Unresectable Hepatocellular Carcinoma. N. Engl. J. Med..

[58] Kim R.D., Chung V., Alese O.B., El-Rayes B.F., Li D., Al-Toubah T.E., Schell M.J., Zhou J.M., Mahipal A., Kim B.H. (2020). A Phase 2 Multi-institutional Study of Nivolumab for Patients With Advanced Refractory Biliary Tract Cancer. JAMA Oncol..

[59] Klein O., Kee D., Nagrial A., Markman B., Underhill C., Michael M., Jackett L., Lum C., Behren A., Palmer J. (2020). Evaluation of Combination Nivolumab and Ipilimumab Immunotherapy in Patients With Advanced Biliary Tract Cancers: Subgroup Analysis of a Phase 2 Nonrandomized Clinical Trial. JAMA Oncol..

[60] O′Reilly E.M., Oh D.Y., Dhani N., Renouf D.J., Lee M.A., Sun W., Fisher G., Hezel A., Chang S.C., Vlahovic G. (2019). Durvalumab With or Without Tremelimumab for Patients With Metastatic Pancreatic Ductal Adenocarcinoma: A Phase 2 Randomized Clinical Trial. JAMA Oncol..

[61] Patel S.P., Othus M., Chae Y.K., Giles F.J., Hansel D.E., Singh P.P., Fontaine A., Shah M.H., Kasi A., Baghdadi T.A. (2020). A Phase II Basket Trial of Dual Anti–CTLA-4 and Anti–PD-1 Blockade in Rare Tumors (DART SWOG 1609) in Patients with Nonpancreatic Neuroendocrine Tumors. Clin. Cancer Res..

[62] Halperin D.M., Liu S., Dasari A., Fogelman D.R., Bhosale P., Mahvash A., Dervin S., Estrella J., Cortazar P., Maru D.M. (2020). A phase II trial of atezolizumab and bevacizumab in patients with advanced, progressive neuroendocrine tumors (NETs). J. Clin. Oncol..

[63] Marcus L., Lemery S.J., Keegan P., Pazdur R. (2019). FDA Approval Summary: Pembrolizumab for the Treatment of Microsatellite Instability-High Solid Tumors. Clin. Cancer Res..

[64] Goodman A.M., Kato S., Bazhenova L., Patel S.P., Frampton G.M., Miller V., Stephens P.J., Daniels G.A., Kurzrock R. (2017). Tumor Mutational Burden as an Independent Predictor of Response to Immunotherapy in Diverse Cancers. Mol. Cancer Ther..

[65] Kulangara K., Hanks D.A., Waldroup S., Peltz L., Shah S., Roach C., Juco J.W., Emancipator K., Stanforth D. (2017). Development of the combined positive score (CPS) for the evaluation of PD-L1 in solid tumors with the immunohistochemistry assay PD-L1 IHC 22C3 pharmDx. J. Clin. Oncol..

[66] Kulangara K., Zhang N., Corigliano E., Guerrero L., Waldroup S., Jaiswal D., Ms M.J., Shah S., Hanks D., Wang J. (2019). Clinical Utility of the Combined Positive Score for Programmed Death Ligand-1 Expression and the Approval of Pembrolizumab for Treatment of Gastric Cancer. Arch. Pathol. Lab. Med..

[67] Salem M.E., Puccini A., Grothey A., Raghavan D., Goldberg R.M., Xiu J., Korn W.M., Weinberg B.A., Hwang J.J., Shields A.F. (2018). Landscape of Tumor Mutation Load, Mismatch Repair Deficiency, and PD-L1 Expression in a Large Patient Cohort of Gastrointestinal Cancers. Mol. Cancer Res. MCR.

[68] Cortes-Ciriano I., Lee S., Park W.Y., Kim T.M., Park P.J. (2017). A molecular portrait of microsatellite instability across multiple cancers. Nat. Commun..

[69] Goumard C., Desbois-Mouthon C., Wendum D., Calmel C., Merabtene F., Scatton O., Praz F. (2017). Low Levels of Microsatellite Instability at Simple Repeated Sequences Commonly Occur in Human Hepatocellular Carcinoma. Cancer Genom. Proteom..

[70] Nakamura Y., Okamoto W., Shitara K., Kojima T., Morizane C., Naito Y., Yuki S., Kagawa Y., Narita Y., Nakashima Y. (2018). Large-scale analyses of tumor mutation burdens (TMBs) across various advanced gastrointestinal (GI) malignancies in the nationwide cancer genome screening project, SCRUM-Japan GI-SCREEN. J. Clin. Oncol..

[71] Hu Z.I., Shia J., Stadler Z.K., Varghese A.M., Capanu M., Salo-Mullen E., Lowery M.A., Diaz L.A., Mandelker D., Yu K.H. (2018). Evaluating Mismatch Repair Deficiency in Pancreatic Adenocarcinoma: Challenges and Recommendations. Clin. Cancer Res..

[72] Kim S.T., Cristescu R., Bass A.J., Kim K.M., Odegaard J.I., Kim K., Liu X.Q., Sher X., Jung H., Lee M. (2018). Comprehensive molecular characterization of clinical responses to PD-1 inhibition in metastatic gastric cancer. Nat. Med..

[73] Doi T., Piha-Paul S.A., Jalal S.I., Saraf S., Lunceford J., Koshiji M., Bennouna J. (2018). Safety and Antitumor Activity of the Anti-Programmed Death-1 Antibody Pembrolizumab in Patients With Advanced Esophageal Carcinoma. J. Clin. Oncol..

[74] Ott P.A., Piha-Paul S.A., Munster P., Pishvaian M.J., van Brummelen E.M.J., Cohen R.B., Gomez-Roca C., Ejadi S., Stein M., Chan E. (2017). Safety and antitumor activity of the anti-PD-1 antibody pembrolizumab in patients with recurrent carcinoma of the anal canal. Ann. Oncol..

[75] Marabelle A., Cassier P.A., Fakih M., Kao S.C.-H., Nielsen D., Italiano A., Guren T., Dongen M.V., Spencer K.R., Bariani G.M. (2020). Pembrolizumab for previously treated advanced anal squamous cell carcinoma: Pooled results from the KEYNOTE-028 and KEYNOTE-158 studies. J. Clin. Oncol..

[76] Marabelle A., Cassier P.A., Fakih M., Guren T.K., Italiano A., Kao S.C.-H., Nielsen D., Ascierto P.A., Bariani G.M., Santoro A. (2020). Pembrolizumab for advanced anal squamous cell carcinoma (ASCC): Results from the multicohort, phase II KEYNOTE-158 study. J. Clin. Oncol..

[77] Bang Y.-J., Ueno M., Malka D., Chung H.C., Nagrial A., Kelley R.K., Piha-Paul S.A., Ros W., Italiano A., Nakagawa K. (2019). Pembrolizumab (pembro) for advanced biliary adenocarcinoma: Results from the KEYNOTE-028 (KN028) and KEYNOTE-158 (KN158) basket studies. J. Clin. Oncol..

[78] Royal R.E., Levy C., Turner K., Mathur A., Hughes M., Kammula U.S., Sherry R.M., Topalian S.L., Yang J.C., Lowy I. (2010). Phase 2 trial of single agent Ipilimumab (anti-CTLA-4) for locally advanced or metastatic pancreatic adenocarcinoma. J. Immunother..

[79] Mehnert J.M., Bergsland E., O′Neil B.H., Santoro A., Schellens J.H.M., Cohen R.B., Doi T., Ott P.A., Pishvaian M.J., Puzanov I. (2020). Pembrolizumab for the treatment of programmed death-ligand 1-positive advanced carcinoid or pancreatic neuroendocrine tumors: Results from the KEYNOTE-028 study. Cancer.

[80] Strosberg J., Mizuno N., Doi T., Grande E., Delord J.P., Shapira-Frommer R., Bergsland E., Shah M., Fakih M., Takahashi S. (2020). Efficacy and Safety of Pembrolizumab in Previously Treated Advanced Neuroendocrine Tumors: Results From the Phase II KEYNOTE-158 Study. Clin. Cancer Res..

[81] Rüdiger Siewert J., Feith M., Werner M., Stein H.J. (2000). Adenocarcinoma of the esophagogastric junction: Results of surgical therapy based on anatomical/topographic classification in 1,002 consecutive patients. Ann. Surg..

[82] The Cancer Genome Atlas Research Network (2014). Comprehensive molecular characterization of gastric adenocarcinoma. Nature.

[83] Grogg K.L., Lohse C.M., Pankratz V.S., Halling K.C., Smyrk T.C. (2003). Lymphocyte-Rich Gastric Cancer: Associations with Epstein-Barr Virus, Microsatellite Instability, Histology, and Survival. Mod. Pathol..

[84] Marrelli D., Polom K., Pascale V., Vindigni C., Piagnerelli R., De Franco L., Ferrara F., Roviello G., Garosi L., Petrioli R. (2016). Strong Prognostic Value of Microsatellite Instability in Intestinal Type Non-cardia Gastric Cancer. Ann. Surg. Oncol..

[85] Mathiak M., Warneke V.S., Behrens H.-M., Haag J., Böger C., Krüger S., Röcken C. (2017). Clinicopathologic Characteristics of Microsatellite Instable Gastric Carcinomas Revisited: Urgent Need for Standardization. Appl. Immunohistochem Mol. Morphol..

[86] Choi Y.Y., Bae J.M., An J.Y., Kwon I.G., Cho I., Shin H.B., Eiji T., Aburahmah M., Kim H.I., Cheong J.H. (2014). Is microsatellite instability a prognostic marker in gastric cancer? A systematic review with meta-analysis. J. Surg. Oncol..

[87] Polom K., Marano L., Marrelli D., De Luca R., Roviello G., Savelli V., Tan P., Roviello F. (2018). Meta-analysis of microsatellite instability in relation to clinicopathological characteristics and overall survival in gastric cancer. Br. J. Surg..

[88] Pietrantonio F., Miceli R., Raimondi A., Kim Y.W., Kang W.K., Langley R.E., Choi Y.Y., Kim K.M., Nankivell M.G., Morano F. (2019). Individual Patient Data Meta-Analysis of the Value of Microsatellite Instability As a Biomarker in Gastric Cancer. J. Clin. Oncol..

[89] Smyth E.C., Wotherspoon A., Peckitt C., Gonzalez D., Hulkki-Wilson S., Eltahir Z., Fassan M., Rugge M., Valeri N., Okines A. (2017). Mismatch Repair Deficiency, Microsatellite Instability, and Survival: An Exploratory Analysis of the Medical Research Council Adjuvant Gastric Infusional Chemotherapy (MAGIC) Trial. JAMA Oncol..

[90] Angell H.K., Lee J., Kim K.M., Kim K., Kim S.T., Park S.H., Kang W.K., Sharpe A., Ogden J., Davenport A. (2019). PD-L1 and immune infiltrates are differentially expressed in distinct subgroups of gastric cancer. Oncoimmunology.

[91] Bang Y.J., Van Cutsem E., Fuchs C.S., Ohtsu A., Tabernero J., Ilson D.H., Hyung W.J., Strong V.E., Goetze T.O., Yoshikawa T. (2019). KEYNOTE-585: Phase III study of perioperative chemotherapy with or without pembrolizumab for gastric cancer. Future Oncol..

[92] Herbst R.S., Arkenau H.-T., Santana-Davila R., Calvo E., Paz-Ares L., Cassier P.A., Bendell J., Penel N., Krebs M.G., Martin-Liberal J. (2019). Ramucirumab plus pembrolizumab in patients with previously treated advanced non-small-cell lung cancer, gastro-oesophageal cancer, or urothelial carcinomas (JVDF): A multicohort, non-randomised, open-label, phase 1a/b trial. Lancet Oncol..

[93] Rha S.Y., Lee C.-K., Kim H.S., Kang B., Jung M., Bae W.K., Koo D.-H., Shin S.-J., Jeung H.-C., Zang D.Y. (2020). Targeting HER2 in combination with anti-PD-1 and chemotherapy confers a significant tumor shrinkage of gastric cancer: A multi-institutional phase Ib/II trial of first-line triplet regimen (pembrolizumab, trastuzumab, chemotherapy) for HER2-positive advanced gastric cancer (AGC). J. Clin. Oncol..

[94] Kankeu Fonkoua L.A., Chakrabarti S., Sonbol M.B., Kasi P.M., Starr J.S., Liu A.J., Bois M.C., Pitot H.C., Chandrasekharan C., Ross H.J. (2020). Enhanced efficacy of anti-VEGFR2/taxane therapy after progression on immune checkpoint inhibition (ICI) in patients (pts) with metastatic gastroesophageal adenocarcinoma (mGEA). J. Clin. Oncol..

[95] (2017). Integrated genomic characterization of oesophageal carcinoma. Nature.

[96] Imamura Y., Watanabe M., Toihata T., Takamatsu M., Kawachi H., Haraguchi I., Ogata Y., Yoshida N., Saeki H., Oki E. (2019). Recent Incidence Trend of Surgically Resected Esophagogastric Junction Adenocarcinoma and Microsatellite Instability Status in Japanese Patients. Digestion.

[97] Neugut A.I., Jacobson J.S., Suh S., Mukherjee R., Arber N. (1998). The epidemiology of cancer of the small bowel. Cancer Epidemiol. Biomark. Prev..

[98] Bilimoria K.Y., Bentrem D.J., Wayne J.D., Ko C.Y., Bennett C.L., Talamonti M.S. (2009). Small bowel cancer in the United States: Changes in epidemiology, treatment, and survival over the last 20 years. Ann. Surg..

[99] Young J.I., Mongoue-Tchokote S., Wieghard N., Mori M., Vaccaro G.M., Sheppard B.C., Tsikitis V.L. (2016). Treatment and Survival of Small-bowel Adenocarcinoma in the United States: A Comparison With Colon Cancer. Dis. Colon rectum.

[100] Koornstra J.J., Kleibeuker J.H., Vasen H.F. (2008). Small-bowel cancer in Lynch syndrome: Is it time for surveillance?. Lancet Oncol..

[101] Schrock A.B., Devoe C.E., McWilliams R., Sun J., Aparicio T., Stephens P.J., Ross J.S., Wilson R., Miller V.A., Ali S.M. (2017). Genomic Profiling of Small-Bowel Adenocarcinoma. JAMA Oncol..

[102] Ribic C.M., Sargent D.J., Moore M.J., Thibodeau S.N., French A.J., Goldberg R.M., Hamilton S.R., Laurent-Puig P., Gryfe R., Shepherd L.E. (2003). Tumor microsatellite-instability status as a predictor of benefit from fluorouracil-based adjuvant chemotherapy for colon cancer. N. Engl. J. Med..

[103] Bertagnolli M.M., Redston M., Compton C.C., Niedzwiecki D., Mayer R.J., Goldberg R.M., Colacchio T.A., Saltz L.B., Warren R.S. (2011). Microsatellite instability and loss of heterozygosity at chromosomal location 18q: Prospective evaluation of biomarkers for stages II and III colon cancer--a study of CALGB 9581 and 89803. J. Clin. Oncol..

[104] Sargent D.J., Marsoni S., Monges G., Thibodeau S.N., Labianca R., Hamilton S.R., French A.J., Kabat B., Foster N.R., Torri V. (2010). Defective mismatch repair as a predictive marker for lack of efficacy of fluorouracil-based adjuvant therapy in colon cancer. J. Clin. Oncol..

[105] Latham A., Reidy D.L., Segal N.H., Yaeger R., Ganesh K., Connell L.C., Kemeny N.E., Shia J., Hechtman J.F., Srinivasan P. (2019). Characterization and clinical outcomes of mismatch repair deficient (dMMR) small bowel adenocarcinoma (SBA). J. Clin. Oncol..

[106] Vanoli A., Grillo F., Guerini C., Neri G., Arpa G., Klersy C., Nesi G., Giuffrida P., Sampietro G., Ardizzone S. (2021). Prognostic Role of Mismatch Repair Status, Histotype and High-Risk Pathologic Features in Stage II Small Bowel Adenocarcinomas. Ann. Surg. Oncol..

[107] Giuffrida P., Arpa G., Grillo F., Klersy C., Sampietro G., Ardizzone S., Fociani P., Fiocca R., Latella G., Sessa F. (2020). PD-L1 in small bowel adenocarcinoma is associated with etiology and tumor-infiltrating lymphocytes, in addition to microsatellite instability. Mod. Pathol..

[108] Zhu M., Zhang H., Foster N.R., Dong H., Bekaii-Saab T.S., Jaszewski B.L., Boland P.M., Overman M.J., Pedersen K., McWilliams R.R. (2020). Abstract 4467: Bim and CX3CR1/granzyme B in circulating CD8+ T cells are predictive biomarkers for PD-1 blockade therapy. Cancer Res..

[109] Network N.C.C. Small Bowel Adenocarcinoma (NCCN Guidelines Version 2.2020). https://www.nccn.org/professionals/physician_gls/pdf/small_bowel.pdf.

[110] (2012). Human papillomavirus-associated cancers—United States, 2004–2008. MMWR. Morb. Mortal. Wkly. Rep..

[111] Daling J.R., Madeleine M.M., Johnson L.G., Schwartz S.M., Shera K.A., Wurscher M.A., Carter J.J., Porter P.L., Galloway D.A., McDougall J.K. (2004). Human papillomavirus, smoking, and sexual practices in the etiology of anal cancer. Cancer.

[112] Serup-Hansen E., Linnemann D., Skovrider-Ruminski W., Høgdall E., Geertsen P.F., Havsteen H. (2014). Human Papillomavirus Genotyping and p16 Expression As Prognostic Factors for Patients With American Joint Committee on Cancer Stages I to III Carcinoma of the Anal Canal. J. Clin. Oncol..

[113] Hu W.H., Miyai K., Cajas-Monson L.C., Luo L., Liu L., Ramamoorthy S.L. (2015). Tumor-infiltrating CD8(+) T lymphocytes associated with clinical outcome in anal squamous cell carcinoma. J. Surg. Oncol..

[114] Balermpas P., Martin D., Wieland U., Rave-Fränk M., Strebhardt K., Rödel C., Fokas E., Rödel F. (2017). Human papilloma virus load and PD-1/PD-L1, CD8+ and FOXP3 in anal cancer patients treated with chemoradiotherapy: Rationale for immunotherapy. Oncoimmunology.

[115] Luen S.J., Salgado R., Fox S., Savas P., Eng-Wong J., Clark E., Kiermaier A., Swain S.M., Baselga J., Michiels S. (2017). Tumour-infiltrating lymphocytes in advanced HER2-positive breast cancer treated with pertuzumab or placebo in addition to trastuzumab and docetaxel: A retrospective analysis of the CLEOPATRA study. Lancet Oncol..

[116] Ferris R.L., Blumenschein G., Fayette J., Guigay J., Colevas A.D., Licitra L., Harrington K., Kasper S., Vokes E.E., Even C. (2016). Nivolumab for Recurrent Squamous-Cell Carcinoma of the Head and Neck. N. Engl. J. Med..

[117] Bauml J., Seiwert T.Y., Pfister D.G., Worden F., Liu S.V., Gilbert J., Saba N.F., Weiss J., Wirth L., Sukari A. (2017). Pembrolizumab for Platinum- and Cetuximab-Refractory Head and Neck Cancer: Results From a Single-Arm, Phase II Study. J. Clin. Oncol..

[118] Ryerson A.B., Eheman C.R., Altekruse S.F., Ward J.W., Jemal A., Sherman R.L., Henley S.J., Holtzman D., Lake A., Noone A.M. (2016). Annual Report to the Nation on the Status of Cancer, 1975–2012, featuring the increasing incidence of liver cancer. Cancer.

[119] Sanyal A., Poklepovic A., Moyneur E., Barghout V. (2010). Population-based risk factors and resource utilization for HCC: US perspective. Curr. Med Res. Opin..

[120] Akinyemiju T., Abera S., Ahmed M., Alam N., Alemayohu M.A., Allen C., Al-Raddadi R., Alvis-Guzman N., Amoako Y., Artaman A. (2017). The Burden of Primary Liver Cancer and Underlying Etiologies From 1990 to 2015 at the Global, Regional, and National Level: Results From the Global Burden of Disease Study 2015. JAMA Oncol..

[121] Marrero J.A., Kulik L.M., Sirlin C.B., Zhu A.X., Finn R.S., Abecassis M.M., Roberts L.R., Heimbach J.K. (2018). Diagnosis, Staging, and Management of Hepatocellular Carcinoma: 2018 Practice Guidance by the American Association for the Study of Liver Diseases. Hepatology.

[122] Llovet J.M., Real M.I., Montaña X., Planas R., Coll S., Aponte J., Ayuso C., Sala M., Muchart J., Solà R. (2002). Arterial embolisation or chemoembolisation versus symptomatic treatment in patients with unresectable hepatocellular carcinoma: A randomised controlled trial. Lancet.

[123] Chen M.S., Li J.Q., Zheng Y., Guo R.P., Liang H.H., Zhang Y.Q., Lin X.J., Lau W.Y. (2006). A prospective randomized trial comparing percutaneous local ablative therapy and partial hepatectomy for small hepatocellular carcinoma. Ann. Surg..

[124] Kulik L.M., Carr B.I., Mulcahy M.F., Lewandowski R.J., Atassi B., Ryu R.K., Sato K.T., Benson A., Nemcek A.A., Gates V.L. (2008). Safety and efficacy of 90Y radiotherapy for hepatocellular carcinoma with and without portal vein thrombosis. Hepatology.

[125] Malagari K., Pomoni M., Kelekis A., Pomoni A., Dourakis S., Spyridopoulos T., Moschouris H., Emmanouil E., Rizos S., Kelekis D. (2010). Prospective randomized comparison of chemoembolization with doxorubicin-eluting beads and bland embolization with BeadBlock for hepatocellular carcinoma. Cardiovasc. Interv. Radiol..

[126] Bush D.A., Smith J.C., Slater J.D., Volk M.L., Reeves M.E., Cheng J., Grove R., de Vera M.E. (2016). Randomized Clinical Trial Comparing Proton Beam Radiation Therapy with Transarterial Chemoembolization for Hepatocellular Carcinoma: Results of an Interim Analysis. Int. J. Radiat. Oncol. Biol. Phys..

[127] Mazzaferro V., Regalia E., Doci R., Andreola S., Pulvirenti A., Bozzetti F., Montalto F., Ammatuna M., Morabito A., Gennari L. (1996). Liver transplantation for the treatment of small hepatocellular carcinomas in patients with cirrhosis. New Engl. J. Med..

[128] Mazzaferro V., Chun Y.S., Poon R.T., Schwartz M.E., Yao F.Y., Marsh J.W., Bhoori S., Lee S.G. (2008). Liver transplantation for hepatocellular carcinoma. Ann. Surg. Oncol..

[129] Llovet J.M., Ricci S., Mazzaferro V., Hilgard P., Gane E., Blanc J.-F., de Oliveira A.C., Santoro A., Raoul J.-L., Forner A. (2008). Sorafenib in Advanced Hepatocellular Carcinoma. N. Engl. J. Med..

[130] Kudo M., Finn R.S., Qin S., Han K.H., Ikeda K., Piscaglia F., Baron A., Park J.W., Han G., Jassem J. (2018). Lenvatinib versus sorafenib in first-line treatment of patients with unresectable hepatocellular carcinoma: A randomised phase 3 non-inferiority trial. Lancet.

[131] Ringelhan M., Pfister D., O′Connor T., Pikarsky E., Heikenwalder M. (2018). The immunology of hepatocellular carcinoma. Nat. Immunol..

[132] Bruix J., Qin S., Merle P., Granito A., Huang Y.H., Bodoky G., Pracht M., Yokosuka O., Rosmorduc O., Breder V. (2017). Regorafenib for patients with hepatocellular carcinoma who progressed on sorafenib treatment (RESORCE): A randomised, double-blind, placebo-controlled, phase 3 trial. Lancet.

[133] Abou-Alfa G.K., Meyer T., Cheng A.-L., El-Khoueiry A.B., Rimassa L., Ryoo B.-Y., Cicin I., Merle P., Chen Y., Park J.-W. (2018). Cabozantinib in Patients with Advanced and Progressing Hepatocellular Carcinoma. N. Engl. J. Med..

[134] Zhu A.X., Kang Y.K., Yen C.J., Finn R.S., Galle P.R., Llovet J.M., Assenat E., Brandi G., Pracht M., Lim H.Y. (2019). Ramucirumab after sorafenib in patients with advanced hepatocellular carcinoma and increased α-fetoprotein concentrations (REACH-2): A randomised, double-blind, placebo-controlled, phase 3 trial. Lancet Oncol..

[135] Valle J., Wasan H., Palmer D.H., Cunningham D., Anthoney A., Maraveyas A., Madhusudan S., Iveson T., Hughes S., Pereira S.P. (2010). Cisplatin plus Gemcitabine versus Gemcitabine for Biliary Tract Cancer. N. Engl. J. Med..

[136] Lamarca A., Palmer D.H., Wasan H.S., Ross P.J., Ma Y.T., Arora A., Falk S., Gillmore R., Wadsley J., Patel K. (2019). ABC-06 | A randomised phase III, multi-centre, open-label study of active symptom control (ASC) alone or ASC with oxaliplatin/5-FU chemotherapy (ASC+mFOLFOX) for patients (pts) with locally advanced/metastatic biliary tract cancers (ABC) previously-treated with cisplatin/gemcitabine (CisGem) chemotherapy. J. Clin. Oncol..

[137] Doebele R.C., Drilon A., Paz-Ares L., Siena S., Shaw A.T., Farago A.F., Blakely C.M., Seto T., Cho B.C., Tosi D. (2020). Entrectinib in patients with advanced or metastatic NTRK fusion-positive solid tumours: Integrated analysis of three phase 1-2 trials. Lancet Oncol..

[138] Drilon A., Laetsch T.W., Kummar S., DuBois S.G., Lassen U.N., Demetri G.D., Nathenson M., Doebele R.C., Farago A.F., Pappo A.S. (2018). Efficacy of Larotrectinib in TRK Fusion–Positive Cancers in Adults and Children. N. Engl. J. Med..

[139] Abou-Alfa G.K., Macarulla T., Javle M.M., Kelley R.K., Lubner S.J., Adeva J., Cleary J.M., Catenacci D.V., Borad M.J., Bridgewater J. (2020). Ivosidenib in IDH1-mutant, chemotherapy-refractory cholangiocarcinoma (ClarIDHy): A multicentre, randomised, double-blind, placebo-controlled, phase 3 study. Lancet Oncol..

[140] Abou-Alfa G.K., Sahai V., Hollebecque A., Vaccaro G., Melisi D., Al-Rajabi R., Paulson A.S., Borad M.J., Gallinson D., Murphy A.G. (2020). Pemigatinib for previously treated, locally advanced or metastatic cholangiocarcinoma: A multicentre, open-label, phase 2 study. Lancet Oncol..

[141] Waddell N., Pajic M., Patch A.M., Chang D.K., Kassahn K.S., Bailey P., Johns A.L., Miller D., Nones K., Quek K. (2015). Whole genomes redefine the mutational landscape of pancreatic cancer. Nature.

[142] Whatcott C.J., Diep C.H., Jiang P., Watanabe A., LoBello J., Sima C., Hostetter G., Shepard H.M., Von Hoff D.D., Han H. (2015). Desmoplasia in Primary Tumors and Metastatic Lesions of Pancreatic Cancer. Clin. Cancer Res..

[143] Ho W.J., Jaffee E.M., Zheng L. (2020). The tumour microenvironment in pancreatic cancer—clinical challenges and opportunities. Nat. Rev. Clin. Oncol..

[144] O′Donnell J.S., Teng M.W.L., Smyth M.J. (2019). Cancer immunoediting and resistance to T cell-based immunotherapy. Nat. Rev. Clin. Oncol..

[145] Osipov A., Zaidi N., Laheru D.A. (2019). Dual Checkpoint Inhibition in Pancreatic Cancer: Revealing the Limitations of Synergy and the Potential of Novel Combinations. JAMA Oncol..

[146] Regalla D.K.R., Jacob R., Manne A., Paluri R.K. (2019). Therapeutic options for ampullary carcinomas. A review. Oncol. Rev..

[147] Xue Y., Balci S., Aydin Mericoz C., Taskin O.C., Jiang H., Pehlivanoglu B., Muraki T., Memis B., Saka B., Kim G.E. (2020). Frequency and clinicopathologic associations of DNA mismatch repair protein deficiency in ampullary carcinoma: Routine testing is indicated. Cancer.

[148] Ruemmele P., Dietmaier W., Terracciano L., Tornillo L., Bataille F., Kaiser A., Wuensch P.H., Heinmoeller E., Homayounfar K., Luettges J. (2009). Histopathologic features and microsatellite instability of cancers of the papilla of vater and their precursor lesions. Am. J. Surg. Pathol..

[149] Nagtegaal I.D., Odze R.D., Klimstra D., Paradis V., Rugge M., Schirmacher P., Washington K.M., Carneiro F., Cree I.A. (2020). The 2019 WHO classification of tumours of the digestive system. Histopathology.

[150] Strosberg J.R., Fine R.L., Choi J., Nasir A., Coppola D., Chen D.T., Helm J., Kvols L. (2011). First-line chemotherapy with capecitabine and temozolomide in patients with metastatic pancreatic endocrine carcinomas. Cancer.

[151] Sahnane N., Furlan D., Monti M., Romualdi C., Vanoli A., Vicari E., Solcia E., Capella C., Sessa F., La Rosa S. (2015). Microsatellite unstable gastrointestinal neuroendocrine carcinomas: A new clinicopathologic entity. Endocr.-Relat. Cancer.

[152] Maggio I., Manuzzi L., Lamberti G., Ricci A.D., Tober N., Campana D. (2020). Landscape and Future Perspectives of Immunotherapy in Neuroendocrine Neoplasia. Cancers.

[153] Yao J.C., Strosberg J., Fazio N., Pavel M.E., Ruszniewski P., Bergsland E., Li D., Tafuto S., Raj N., Campana D. (2018). Activity & safety of spartalizumab (PDR001) in patients (pts) with advanced neuroendocrine tumors (NET) of pancreatic (Pan), gastrointestinal (GI), or thoracic (T) origin, & gastroenteropancreatic neuroendocrine carcinoma (GEP NEC) who have progressed on prior treatment (Tx). Ann. Oncol..

[154] Strosberg J.R., Mizuno N., Doi T., Grande E., Delord J.-P., Shapira-Frommer R., Bergsland E.K., Shah M.H., Fakih M., Takahashi S. (2019). Pembrolizumab treatment of advanced neuroendocrine tumors: Results from the phase II KEYNOTE-158 study. J. Clin. Oncol..

[155] Schreiber R.D., Old L.J., Smyth M.J. (2011). Cancer immunoediting: Integrating immunity′s roles in cancer suppression and promotion. Science.

[156] Gordon S.R., Maute R.L., Dulken B.W., Hutter G., George B.M., McCracken M.N., Gupta R., Tsai J.M., Sinha R., Corey D. (2017). PD-1 expression by tumour-associated macrophages inhibits phagocytosis and tumour immunity. Nature.

[157] Fares C.M., Van Allen E.M., Drake C.G., Allison J.P., Hu-Lieskovan S. (2019). Mechanisms of Resistance to Immune Checkpoint Blockade: Why Does Checkpoint Inhibitor Immunotherapy Not Work for All Patients?. Am. Soc. Clin. Oncol. Educ. Book.

[158] Kalbasi A., Ribas A. (2020). Tumour-intrinsic resistance to immune checkpoint blockade. Nat. Rev. Immunol..

[159] Zaretsky J.M., Garcia-Diaz A., Shin D.S., Escuin-Ordinas H., Hugo W., Hu-Lieskovan S., Torrejon D.Y., Abril-Rodriguez G., Sandoval S., Barthly L. (2016). Mutations Associated with Acquired Resistance to PD-1 Blockade in Melanoma. New Engl. J. Med..

[160] Shin D.S., Zaretsky J.M., Escuin-Ordinas H., Garcia-Diaz A., Hu-Lieskovan S., Kalbasi A., Grasso C.S., Hugo W., Sandoval S., Torrejon D.Y. (2017). Primary Resistance to PD-1 Blockade Mediated by *JAK1/2* Mutations. Cancer Discov..

[161] Yoon H.H., Sinicrope F.A. (2018). Heterogeneity in the lymphocytic infiltration of deficient DNA mismatch repair colon cancers. Oncotarget.

[162] O′Donnell J.S., Hoefsmit E.P., Smyth M.J., Blank C.U., Teng M.W.L. (2019). The Promise of Neoadjuvant Immunotherapy and Surgery for Cancer Treatment. Clin. Cancer Res..

[163] Kelly R.J., Ajani J.A., Kuzdzal J., Zander T., Van Cutsem E., Piessen G., Mendez G., Feliciano J.L., Motoyama S., LIÈVRE A. (2020). Moehler. Adjuvant nivolumab in resected esophageal or gastroesophageal junction cancer (EC/GEJC) following neoadjuvant chemoradiation therapy (CRT): First results of the CheckMate 577 study. Ann. Oncol..

[164] Borcoman E., Nandikolla A., Long G., Goel S., Tourneau C.L. (2018). Patterns of Response and Progression to Immunotherapy. Am. Soc. Clin. Oncol. Educ. Book.

[165] Queirolo P., Spagnolo F. (2017). Atypical responses in patients with advanced melanoma, lung cancer, renal-cell carcinoma and other solid tumors treated with anti-PD-1 drugs: A systematic review. Cancer Treat. Rev..

[166] Champiat S., Dercle L., Ammari S., Massard C., Hollebecque A., Postel-Vinay S., Chaput N., Eggermont A., Marabelle A., Soria J.C. (2017). Hyperprogressive Disease Is a New Pattern of Progression in Cancer Patients Treated by Anti-PD-1/PD-L1. Clin. Cancer Res..

[167] Kato S., Goodman A., Walavalkar V., Barkauskas D.A., Sharabi A., Kurzrock R. (2017). Hyperprogressors after Immunotherapy: Analysis of Genomic Alterations Associated with Accelerated Growth Rate. Clin. Cancer Res..

[168] Saâda-Bouzid E., Defaucheux C., Karabajakian A., Coloma V.P., Servois V., Paoletti X., Even C., Fayette J., Guigay J., Loirat D. (2017). Hyperprogression during anti-PD-1/PD-L1 therapy in patients with recurrent and/or metastatic head and neck squamous cell carcinoma. Ann. Oncol..

[169] Gibney G.T., Weiner L.M., Atkins M.B. (2016). Predictive biomarkers for checkpoint inhibitor-based immunotherapy. Lancet Oncol..

[170] Spranger S., Spaapen R.M., Zha Y., Williams J., Meng Y., Ha T.T., Gajewski T.F. (2013). Up-regulation of PD-L1, IDO, and T(regs) in the melanoma tumor microenvironment is driven by CD8(+) T cells. Sci. Transl. Med..

[171] Garon E.B., Rizvi N.A., Hui R., Leighl N., Balmanoukian A.S., Eder J.P., Patnaik A., Aggarwal C., Gubens M., Horn L. (2015). Pembrolizumab for the treatment of non-small-cell lung cancer. N. Engl. J. Med..

[172] Postow M.A., Manuel M., Wong P., Yuan J., Dong Z., Liu C., Perez S., Tanneau I., Noel M., Courtier A. (2015). Peripheral T cell receptor diversity is associated with clinical outcomes following ipilimumab treatment in metastatic melanoma. J. Immunother. Cancer.

[173] Ferrucci P.F., Ascierto P.A., Pigozzo J., Del Vecchio M., Maio M., Antonini Cappellini G.C., Guidoboni M., Queirolo P., Savoia P., Mandalà M. (2016). Baseline neutrophils and derived neutrophil-to-lymphocyte ratio: Prognostic relevance in metastatic melanoma patients receiving ipilimumab. Ann. Oncol..

[174] Zeng D.Q., Yu Y.F., Ou Q.Y., Li X.Y., Zhong R.Z., Xie C.M., Hu Q.G. (2016). Prognostic and predictive value of tumor-infiltrating lymphocytes for clinical therapeutic research in patients with non-small cell lung cancer. Oncotarget.

[175] Higgs B.W., Morehouse C., Streicher K., Rebelatto M.C., Steele K., Jin X., Pilataxi F., Brohawn P.Z., Blake-Haskins J.A., Gupta A.K. (2016). Relationship of baseline tumoral IFNγ mRNA and PD-L1 protein expression to overall survival in durvalumab-treated NSCLC patients. J. Clin. Oncol..

[176] Martens A., Wistuba-Hamprecht K., Geukes Foppen M., Yuan J., Postow M.A., Wong P., Romano E., Khammari A., Dreno B., Capone M. (2016). Baseline Peripheral Blood Biomarkers Associated with Clinical Outcome of Advanced Melanoma Patients Treated with Ipilimumab. Clin. Cancer Res..

